# Reassessing *Helicobacter pylori* Eradication Strategies in the Era of Rising Antibiotic Resistance

**DOI:** 10.5152/tjg.2026.25699

**Published:** 2026-02-19

**Authors:** Tuba Yilmaz Yildirım, Osman Cavit Özdoğan

**Affiliations:** Department of Gastroenterology, Marmara University School of Medicine, İstanbul, Türkiye

Dear Editor,

We read with great interest the article by Unler et al^1^ entitled “Best Treatment Options for Severe Helicobacter pylori Infections.” *Helicobacter pylori* (*H. pylori*) remains one of the most widespread bacterial infections worldwide, leading to chronic gastritis, peptic ulcer disease, gastric adenocarcinoma, unexplained iron-deficiency anemia, and vitamin B12 deficiency.^2^ The authors compared bismuth-containing quadruple therapy (BQT), bismuth-containing sequential therapy with clarithromycin (BSTC), and bismuth-containing sequential therapy with levofloxacin (BSTL), while also examining the impact of gastric *H. pylori* density on eradication outcomes. Their finding that BSTL yielded the highest eradication rate, particularly in high-density infection, provides valuable insight for clinical practice in Türkiye.

However, several methodological and interpretative considerations warrant further discussion, especially concerning current antibiotic resistance rates in Türkiye and international guideline recommendations. The authors acknowledge the absence of antimicrobial susceptibility testing as a limitation. In a country such as Türkiye, where antibiotic resistance rates are known to be high, this limitation becomes particularly critical when interpreting comparative eradication outcomes. A recent Turkish meta-analysis reported resistance rates of 26.7% for clarithromycin, 28.4% for metronidazole, and 19.6% for levofloxacin, with much lower resistance to amoxicillin (1.3%) and tetracycline (0.7%).^3^ These resistance patterns have remained consistently high over recent years, while resistance to amoxicillin and tetracycline has remained low or even decreased.^4^ In this context, higher eradication rates observed with BSTL may reflect background resistance patterns rather than intrinsic superiority of the regimen itself. Therefore, without susceptibility testing, the conclusion that BSTL represents the most effective first-line option may be prone to overinterpretation.

The authors demonstrated a progressive decline in eradication success with increasing *H. pylori* density for both BQT and BSTC, whereas BSTL maintained relatively stable outcomes even in patients with severe colonization. High bacterial load has been shown to contribute to eradication failure through an inoculum effect, potentially limiting antibiotic penetration and effectiveness.^5^ These findings suggest that patients with severe colonization may require more potent or optimized regimens. Nevertheless, in the absence of susceptibility testing, it remains unclear whether treatment failure in high-density infections is driven primarily by bacterial load or by coexisting resistance to specific antibiotics. This distinction is clinically important when translating study findings into empiric treatment recommendations.

In this context, the conclusion that BSTL may be the best option for treating *H. pylori* infections in first-line treatment in Türkiye should be interpreted cautiously. Current international guidelines including the American College of Gastroenterology guidelines, BQT is advised as the empiric first-line regimen for patients not previously treated for *H. pylori*, as it generally achieves eradication rates above 85%. In areas of high clarithromycin resistance (≥15%), 14-day concomitant therapy or 14-day BQT is considered the most suitable as first-line regimen.^6^ Levofloxacin-based regimens are generally reserved for rescue or second-line therapy rather than empiric first-line use. Consequently, the study’s findings should not be interpreted as supporting a change in first-line treatment strategy in Türkiye.

The authors briefly mention vonoprazan as a newer, more potent acid suppressant that might improve eradication rates. Recent meta-analyses have demonstrated that vonoprazan-based dual therapy with high-dose amoxicillin achieves high eradication rates with favorable tolerability.^7^ However, its availability and real-world applicability remain limited in many regions, including Türkiye. Therefore, vonoprazan-based regimens don’t influence the interpretation of the results presented by Ünler et al.

In summary, the study by Ünler et al^1^ provides valuable real-world data on the comparative efficacy of bismuth-containing regimens and highlights the clinical relevance of bacterial density. Nevertheless, due to the absence of antimicrobial susceptibility testing and the high background resistance rates in Türkiye, the apparent superiority of BSTL should be interpreted cautiously. Fourteen-day BQT remains the most guideline-concordant empiric first-line option, while levofloxacin-based sequential regimens such as BSTL may be more appropriately considered in selected cases or as second-line therapy.

## Data Availability

The data that support the findings of this study are available on request from the corresponding author.
